# A scoping review to examine health care professionals’ experiences as family caregivers

**DOI:** 10.1371/journal.pone.0308657

**Published:** 2025-01-22

**Authors:** Kristina M. Kokorelias, Nira Rittenberg, Orianna Scali, Suzanne Smith-Bayley, Monique A. M. Gignac, Gary Naglie, Jessica Sheirs, Jill I. Cameron

**Affiliations:** 1 Department of Geriatric Medicine, Sinai Health System and University Health Network, Toronto, ON, Canada; 2 Department of Occupational Science & Occupational Therapy, Temerty Faculty of Medicine, University of Toronto, Toronto, ON, Canada; 3 Rehabilitation Sciences Institute, Temerty Faculty of Medicine, University of Toronto, Toronto, ON, Canada; 4 March of Dimes Canada, Toronto, ON, Canada; 5 Institute for Work & Health, Toronto, ON, Canada; 6 Department of Medicine, University of Toronto, Toronto, ON, Canada; 7 Institute of Health Policy, Management and Evaluation, University of Toronto, Toronto, ON, Canada; 8 Rotman Research Institute, Baycrest Health Sciences, Toronto, ON, Canada; 9 Dalla Lana School of Public Health, Institute for Work and Health, University of Toronto, Toronto, ON, Canada; Bangladesh Open University, BANGLADESH

## Abstract

**Background:**

Health and social care systems must confront the challenge of supporting a growing elderly population and their caregivers. Family caregivers who are healthcare professionals are part of this context, but their caregiving experiences remain unclear.

**Objective:**

This scoping review explored the experiences of healthcare professionals who are also family caregivers for older adults.

**Methodology:**

A scoping review methodology identified and summarized pertinent studies. Searches were conducted in Medline, Embase, PsycINFO, CINAHL, and AgeLine. We sought articles published from each journals’ inception to October 19, 2023. Inclusion criteria were English-language studies about healthcare professionals caring for older adult family members. Diverse research designs were included. Data were extracted and synthesized according to key themes.

**Results:**

The review included 19 studies, highlighting four themes. Studies were published between 1994 and 2019, with most studies published before 2017. The overarching theme was "Expectations," where healthcare professional family caregivers faced multifaceted expectations from themselves, their families, and the healthcare system. Expectations also highlights the dual role of participants as both caregivers and healthcare professionals This complex interplay led to a theme related to personal consequences, including stress, guilt, and potential burnout, but also positive aspects of the dual roles. Studies described how dual roles could enhance the quality-of-care healthcare professional caregivers provided to their family members. Finally, support needs were identified, emphasizing the importance of workplace accommodations and support from the healthcare system and peers.

**Conclusions:**

The experiences of healthcare professional family caregivers are shaped by unique expectations, resulting in both positive and negative consequences. The support needs of this group are multifaceted, requiring workplace accommodations and tailored support within the healthcare system. Further research is needed to delve deeper into the nuances of their experiences and develop targeted interventions to alleviate the stress and challenges they face in their dual roles. Understanding the evolving needs of healthcare professional family caregivers over time can inform support strategies along the caregiving trajectory.

## Introduction

Globally, health systems are challenged with providing care to more people, as the baby boomer generation begins to reach older adulthood. Consequently, there is an increase in the number of older adults (individuals ≥ 65 years of age) requiring care due to age-related conditions (e.g., hearing loss, chronic illness, functional impairment) [1]. The rise in older adults requiring care has increased the number of family caregivers (caregivers) who provide a range of care tasks as they support older adults living with illness or disability [2]. A large proportion of the caregiving population are adult children who provide care to a parent, step-parent or parent-in-law [3]. Adult children caregivers were found to experience caregiving differently than other types of caregivers (e.g., spousal caregivers) [3, 4]. Supporting family caregivers in their role can help sustain the ability of older adults to age at home [5].

The nature of caregiving can be complex for adult children because of the variety of responsibilities, as well as the obligations and meaningful occupations they have outside of their caregiving role [4]. For example, numerous adult children caregivers have reported balancing the care of their parent with the care they provide to their own children [6, 7]. Similarly, many adult children caregivers are also balancing caregiving duties with paid employment [8, 9]. Consequently, adult children caregivers who are in paid employment [10] and/or care for children were found to have worse health and well-being than caregivers not balancing multiple occupational roles [7, 11]. Thus, adult children balancing paid employment and caring for an aging parent may have unique support needs.

Many caregivers, including healthcare professionals, often lack the necessary preparation and support when they begin their caregiving roles. Most caregivers are not equipped with the necessary knowledge, skills, and training when they begin their caregiving role, which can result in a lack of preparation and stress [12–14]. Health care professionals often become caregivers to their aging parents because they are seen as the “health professional in the family,” with expertise and preparedness ([15] p. 384). As such, healthcare professionals often have the dual role of clinical practice and family caregiver. In the context of the COVID-19 pandemic, the challenges of this dual role may be amplified given the increase in psychological distress associated with changes in caregiving and work-related tasks and availability of support [16–18]. Given that strategies to support working caregivers have been considered a priority for ensuring the sustainability of health care systems [19], it is important to consider the perspectives and needs of healthcare professionals who are also family caregivers.

Despite the large number of healthcare professionals who are caregivers to aging parents, much of the caregiving literature has excluded their perspectives and experiences [20] or have not considered healthcare professionals as family caregivers (i.e., literature including them as only healthcare professionals) [21]. As such, literature reviews have often explored the experiences of caregivers without considering that they could be both a caregiver and healthcare professional [22, 23]. We have not been able to identify any published literature reviews that aim to understand the experiences or support needs of health care professionals who are also family caregivers. Considering this gap in the literature, we undertook a scoping review to synthesize the available research studies examining the experiences of health care professionals as caregivers to inform future caregiving research. We chose to focus on caregivers to older adults to address the specific challenges and experiences faced by this demographic, given the unique caregiving dynamics associated with aging populations. We also aimed to characterize the methodological quality of this literature to inform future research.

## Methods

### Design

We followed the scoping review framework by Arksey and O’Malley [24] as advanced by Levac et al. [25]. Stages of the scoping review process were: (i) identify the research question, (ii) identify relevant studies, (iii) select studies, (iv) chart the data and (v) collate, summarise and report the results ([24] p. 22). We did not publish a protocol prior to commencing the study.

### Step 1: Identify the research question

We asked: "What are the experiences of registered healthcare professionals who are caregivers to older adult family members?” We defined older adult family members to encompass kin and non-kin significant others. Family caregiving included tasks that support the care or daily activities of older adults. The term "registered healthcare professional" was used to refer to individuals who have obtained formal education and formal registration or licensure in their respective healthcare professions from their official licensing bodies. This includes professionals such as nurses, physicians, physiotherapists, occupational therapists, bioethicists and pharmacists who are required to be registered or licensed to practice. Thus, we excluded some groups of paid caregivers, such as aides, personal support workers and nursing assistants as these individuals may not require formal licensure or registration to practice.

### Step 2: Identify relevant studies

A rehabilitation sciences librarian (JS) prepared the search strategy in consultation with the research team using the following databases: OVID Medline (1946 to 2023, including Epub Ahead of Print, and In Process & Other Non-Indexed Citations), OVID Embase (1947 to 2023), OVID PsycINFO (1806 to 2023), EBSCO CINAHL Plus with Full Text (1981 to 2023) and EBSCO AgeLine (1966 to 2023). Searches were run on October 19, 2023. ***See S1 File.*** Articles were imported into EndNote software and duplicates removed by the first author [26]. A search of grey literature was not included.

### Step 3: Select studies

The review comprised two stages: initial screening of titles and abstracts and full-text screening using Covidence systematic review software [27]. Titles and abstracts were independently screened by two team members. Then, two reviewers independently assessed the full text articles for inclusion. The entire research team was involved in the review process. Disagreements between reviewers were resolved through discussion with a third team member. Finally, the reference lists of included articles were scanned for potential additional articles.

Inclusion criteria were: (1) study focused on any family involvement by a health care professional in the care of an older adult family member; (2) published peer-reviewed studies that used quantitative, qualitative, or mixed methods designs; and (3) English language. Exclusion criteria were: (1) studies that focused on caregiving of children or adolescents and (2) articles used for teaching purposes (e.g., hypothetical case studies).

### Step 4: Charting the data

Data were extracted and synthesized using an Excel data abstraction form [24]. This included study details (e.g., research design, objectives), study characteristics (e.g., study setting), healthcare professional type, patient and caregiver characteristics (e.g., demographics), caregiver involvement, identified needs, and key findings. The data extraction form underwent pilot testing with four reviewers (SA, CE, OS, KMK) extracting data from the same article. Data were extracted from the remaining studies by three reviewers (SA, CE, OS), with KMK ensuring accuracy through period spot-checking.

The included literature consisted mostly of qualitative study designs. We therefore assessed study quality and methodological soundness using the Joanna Briggs Institute’s (JBI) checklist for qualitative research. Two authors (KMK and OS) independently applied each criterion to the included studies. Inter-rater reliability for quality assessments was evaluated using Cohen’s kappa [28]. We conducted the quality assessment not only to assesses the methodological rigour of the included studies but also to provide insights for guiding future rigorous research in this area. While it deviates from Arksey and O’Malley’s protocol [24], quality assessments have become integral to scoping reviews, serving to enhance the credibility of the findings and inform subsequent research endeavors.

### Step 5: Data analysis

The authors quantified the abstracted data using frequencies for the following variables: year of publication, study period, geographic region and study design. Data were then analyzed using a modified thematic analysis approach [29, 30]. The extracted data were collated to identify themes within the data. A modified thematic analysis technique was utilized, including strategies of scrutinizing, and sorting the extracted data according to crucial nuances of the data. First, the research team reviewed all of the charted data. Data analysis for mixed methods studies involved capturing both quantitative results and qualitative findings, if applicable, to provide a comprehensive overview of their contributions to the scoping review objectives. Next, through a series of discussions, the research team developed a tentative codebook (**see S2 File).** These codes were applied to the results and discussion sections of all articles. NVivo software was used to help organize the coding process. Next, the research team independently reviewed the coded data and then discussed their initial perceptions through a series of bi-weekly meetings. After the data was coded, the research team analyzed the data within and then across different groups of participants (i.e., across studies with predominately male or female participants and across various professionals e.g., nurses). The research team discussed potential themes until consensus was achieved, and final themes were developed. These themes are presented below.

## Results

**Fig 1:**
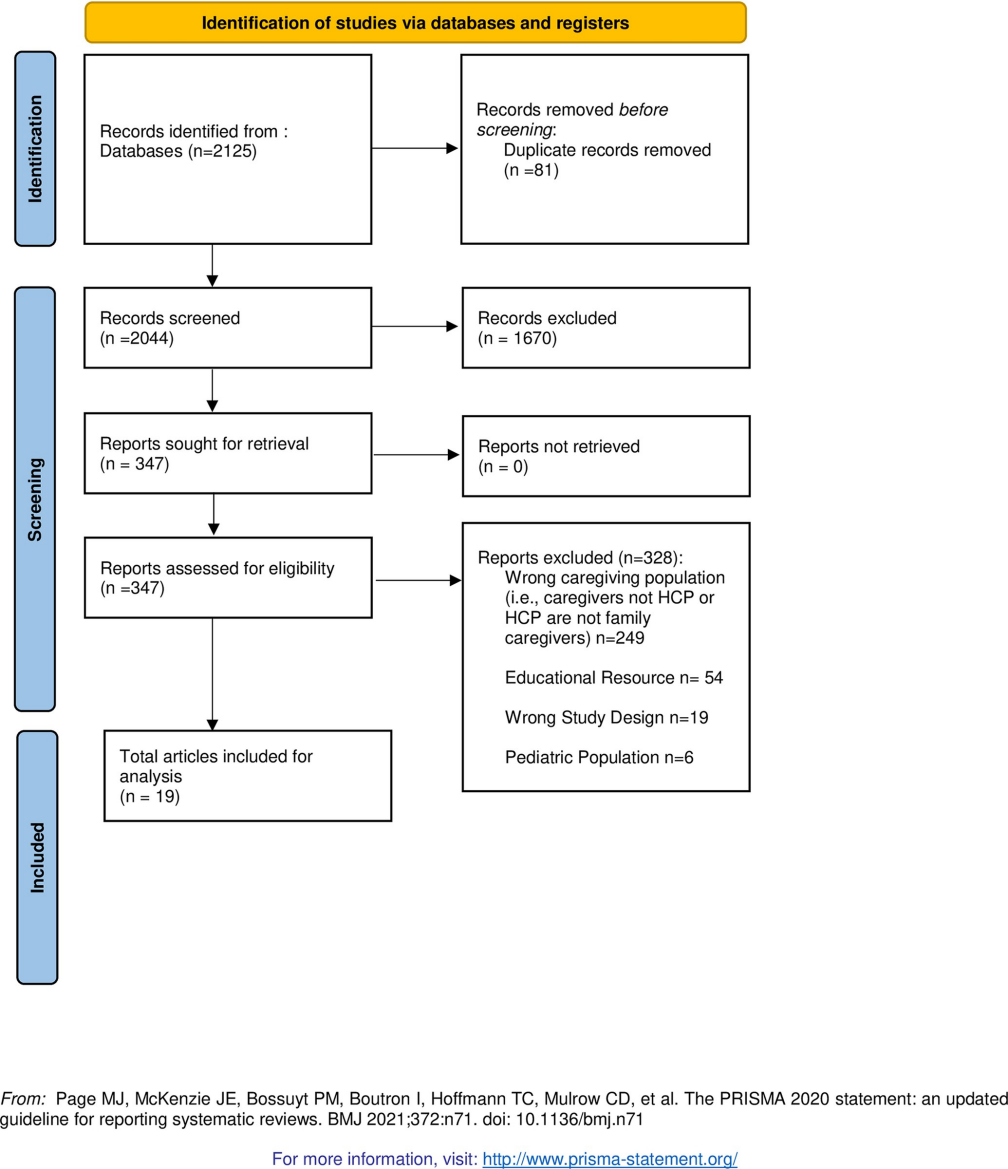
PRISMA 2020 flow diagram for new systematic reviews.

The database search identified 2044 unique records after duplicates were removed. From this, 1670 records were excluded using inclusion and exclusion criteria. A total of 347 full-text articles were assessed for inclusion with 19 studies meeting eligibility criteria (see **Fig 1)**.

### Study characteristics

Studies used qualitative (n = 17/19) and mixed (n = 2/19) methodologies. The mixed methods approach utilized in the two studies incorporated both qualitative and quantitative data collection methods, specifically through the use of interviews and questionnaires. Studies were published from 1994 to 2019, with only 16% (n = 3/19) published from 2017 onwards. Studies were appraised as being of mostly low methodological quality (see **Table 1**). There were a range of geographical regions represented in the studies, with 53% (n = 10/19) conducted in the United States, 26% (n = 5/19) conducted in Canada and two studies conducted in each the United Kingdom (10.5%) and New Zealand (10.5%). Across the 19 studies, the total number of participants was 2,658 (study participants ranged from 1–1399). Qualitative studies had sample sizes from 1–67 and the mixed methods studies had sample sizes that ranged from 1,025–1,399. In the two studies with n = 1, the authors conducted a first-person narrative of their experiences as a case-study. Participants ranged from 18–72 years old [31]. Most articles (n = 14/19, 74%) exclusively included nurses 21% (n = 4/19) exclusively included physicians, and the remaining article (n = 1/19, 5%) included a Bioethicist (see **Table 2)**. Seventy-nine percent of the data sources reported the sex of participants (n = 15/19, 79%) and forty-two percent reported on age (n = 8/19, 45%). Most of the studies included female participants. Over half of the data sources (n = 10/19, 53%) included only females, one study had “mostly females” (n = 1/19, 5%) and three (n = 3/19, 16%) included only males.

**Table 1:** Assessment of study quality.

Author/Title of article	Is there congruity between the stated philosophical perspective and the research methodology?	Is there congruity between the research methodology and the research question or objectives?	Is there congruity between the research methodology and the methods used to collect the data?	Is there congruity between the research methodology and the representation and analysis of the data?	Is there congruity between the research methodology and the interpretation of results?	Is there a statement locating the researcher culturally or theoretically?	Is the influence of the researcher on the research, and vice-versa, addressed?	Are participants, and their voices, adequately represented?	Is the research ethical according to current criteria or, for recent studies, and is there evidence of ethical approval by an appropriate body?	Do the conclusions drawn in the research report flow from the analysis, or interpretation, of the data?
Ross, M M; Rideout, E; Carson, M/Nurses’ work: balancing personal and professional caregiving careers.	Yes	Yes	Yes	Yes	Yes	No	N/A	Yes	N/A	Yes
Klugman, Craig M/Dual roles of the care provider at the end of life: An autoethnography.	N/A	N/A	N/A	N/A	N/A	Yes	N/A	N/A	N/A	Yes
DePasquale, Nicole; Davis, Kelly D; Zarit, Steven H; Moen, Phyllis; Hammer, Leslie B; Almeida, David M/Combining formal and informal caregiving roles: The psychosocial implications of double- and triple-duty care.	Yes	Yes	Yes	Yes	Yes	No	N/A	Yes	N/A	Yes
Wald, Hedy S/Helping My Husband Live and Die.	N/A	N/A	N/A	N/A	N/A	Yes	N/A	N/A	N/A	N/A
Stocker, S/Six tips for caring for aging parents.	Yes	Yes	N/A	N/A	N/A	No	N/A	N/A	N/A	N/A
Simone, C; Seidenschmid, M/Physicians’ role in care of loved ones.	Yes	Yes	N/A	N/A	N/A	N/A	N/A	N/A	N/A	N/A
Fromme, Erik K; Farber, Neil J; Babbott, Stewart F; Pickett, Mary E; Beasley, Brent W/What do you do when your loved one is ill? The line between physician and family member.	Yes	Yes	N/A	N/A	N/A	N/A	N/A	N/A	N/A	N/A
Ward-Griffin, Catherine/Nurses as Caregivers of Elderly Relatives: Negotiating Personal and Professional Boundaries.	Yes	Yes	Yes	Yes	Yes	No	N/A	Yes	N/A	Yes
McConnell, E A/’The daughter is a nurse’.	Yes	Yes	N/A	N/A	N/A	Yes	N/A	N/A	N/A	N/A
Wilson, Kathleen B; Ardoin, Katherine B/When professional and personal worlds meet: nurse as daughter.	Yes	Yes	Yes	Yes	Yes	No	N/A	Yes	N/A	N/A
Quinney, Loretto; Dwyer, Trudy; Chapman, Ysanne/Tensions in the personal world of the nurse family carer: A phenomenological approach.	Yes	Yes	Yes	Yes	Yes	N/A	N/A	No	Yes	Yes
DePasquale, Nicole; Bangerter, Lauren R; Williams, Jessica; Almeida, David M/Certified nursing assistants balancing family caregiving roles: Health care utilization among double- and triple-duty caregivers.	Yes	Yes	Yes	Yes	Yes	N/A	N/A	Yes	N/A	Yes
Anjos, Ana Paula; Ward-Griffin, Catherine; Leipert, Beverly/Understanding gendered expectations and exemptions experienced by male double-duty caregivers: a qualitative secondary analysis.	Yes	Yes	Yes	Yes	Yes	No	N/A	Yes	N/A	Yes
Mills, Jayne; Aubeeluck, Aimee/Nurses’ experiences of caring for their own family members	Yes	Yes	Yes	N/A	N/A	No	N/A	Yes	N/A	Yes
Clendoni, Jill; Walker, Leonie/Juggling nursing and family care.	Yes	Yes	Yes	Yes	Yes	No	N/A	Yes	N/A	Yes
Moskop, John C/Doctor in, and for, the Family?: Physicians Reflect on Care for Loved Ones.	Yes	Yes	Yes	Yes	Yes	N/A	N/A	Yes	N/A	Yes
Cicchelli, Lisa; McLeod, Deborah/Lived experiences of nurses as family caregivers in advanced cancer.	Yes	Yes	Yes	Yes	Yes	N/A	N/A	Yes	N/A	Yes
Clendon J.; Walker L./Nurses as family caregivers—barriers and enablers facing nurses caring for children, parents or both	Yes	Yes	Yes	Yes	Yes	No	N/A	Yes	Yes	Yes
Chen F.M.; Feudtner C.; Rhodes L.A.; Green L.A./Role conflicts of physicians and their family members: Rules but no rulebook	Yes	Yes	Yes	Yes	Yes	N/A	N/A	Yes	N/A	Yes

NOTE: N/A–Not Applicable.

**Table 2:** Characteristics of included peer-reviewed studies and grey literature.

Authors, Country & Year of Publication	Study Design	Objective	Participant Details	Key Conclusions
Anjos, Ana Paula; Ward-Griffin, Catherine; Leipert, Beverly Canada 2012	Qualitative	To explore how gendered expectations and exemptions affect the caregiving experience and personal health of male nurses caring for family members.	Nurses N = 28 Gender/Sex: 100% Male Age: 40–63 years	Male participants mentioned the negative health experiences because of gendered expectations within their role as a nurse and sons. It was concluded that workplace policies, and social services must be looked at again to modify to support the increase in family caregivers.
Chen F.M.; Feudtner C.; Rhodes L.A.; Green L.A. California, United States (USA) 2001	Qualitative	To determine and examine the difficulties that physicians encounter when a family member becomes ill (serious or terminal illness episode within the past 5 years) and uncover the underlying causes.	Physicians N = 8 Gender/Sex: 2 females (25%); 6 males (75%) Age: 43–54 years.	All participants felt that there is a dilemma when it comes to balancing personal as well as professional roles. Caring for father while assuming the role of physician motivated the participants in paying great deal of attention in their care. The study concluded that in order to mitigate conflict when it comes to choosing to become involved in caring for a family member, the study recommends that physicians ask themselves, along with a college about their expectations within their dual role and role conflicts.
Cicchelli, Lisa; McLeod, Deborah Canada 2012	Qualitative	To examine and uncover the experiences of nurses caring for family members who have a diagnosis of advanced cancer.	Nurses N = 5 Gender/Sex: 100% Female Age: ~45 years	Issues arose among the nurses: blurring of boundaries between roles and expectations of care as a nurse and a family caregiver. It is important to gain more insight on dual nursing roles through more in-depth research. Further research will help with understanding the complexities in the role of a nurse taking on the role of s a family caregiver.
Clendon J.; Walker L. New Zealand 2017	Qualitative	To examine the experiences of dual caregiving and nursing responsibilities of nurses in New Zealand to identify potential strategies, employment practices, and policies that can help to retain nurses with these caregiving responsibilities in the workplace.	Nurses N = 28 Gender/Sex: 100% Female Age: 21–70 years	One of the potential impacts of nursing practice is societal and demographic changes related to one’s caregiving role. It is mentioned that managers should accommodate proper support to those nurses working and assuming various responsibilities as a family caregiver. This way, they can cope with both roles and be confident in both roles as well. Also, nurses identified they wanted to work; however, it is a challenge when it comes to lack of support in the workplace setting.
Clendoni, Jill; Walker, Leonie New Zealand 2016	Qualitative	To examine and clearly understand the impact of balancing family care and work on the nurse and workplace.	Nurses N = 67 Gender/Sex: “Mostly female” Age: N/A	Balancing two responsibilities was seen as challenging among the nurses who participated in the study. Change in demographics suggest that there will be increase. Nurses mentioned that having a supportive workplace (boss and colleagues) as well as family members will help in managing both roles (as a nurse and family caregiver).
DePasquale, Nicole; Bangerter, Lauren R; Williams, Jessica; Almeida, David M United Kingdom 2016	Quantitative	To explore the health care utilization among certified nursing assistants (CNAs) working in nursing homes, the majority of whom occupy double- and triple-duty caregiving roles	CNAs who participated: N = 1,025 Gender/Sex: NR Age: NR	Certified Nursing Assistants (CNAs) who provide informal care for older adults seems to have higher acute care visit rates. Further research needs to be done to see how double as well as triple duty caregivers maintain their responsibility and health while giving constant caregiving.
DePasquale, Nicole; Davis, Kelly D; Zarit, Steven H; Moen, Phyllis; Hammer, Leslie B; Almeida, David M United States (USA) 2016	Mixed methods	Women who combine formal and informal caregiving roles represent a unique, understudied population. In the literature, healthcare employees who simultaneously provide unpaid elder care at home have been referred to as double- duty caregivers. The present study broadens this perspective by examining the psychosocial implications of double-duty childcare (childcare only), double-duty elder care (elder care only), and triple-duty care (both childcare and elder care or “sandwiched” care).	Total Sample Size: N = 1399 (67%) held positions as certified nursing assistants (CNA) and registered nurses or licensed practical nurses (28%). Gender/Sex: 100% Female Age: 18–72	Findings from this study suggest that when comparing to employees balancing elder and sandwiched care roles, double duty child caregivers experience less stressors in terms of primary stressors. But this group showed higher secondary stressors in terms of family-related strains relative to nonfamily caregivers.
Fromme, Erik K; Farber, Neil J; Babbott, Stewart F; Pickett, Mary E; Beasley, Brent W United States (USA) 2008	Qualitative	To describe the first-person narratives from physicians involved in the care of family members.	Physicians N = 5 Gender/Sex: NR Age: NR	Family members are meant to be trustworthy which means it is more accessible to the patient. But there are risks when someone has family caregiver as physician: limited self awareness surrounding the care they provide to the family member. Physician should step back and ask themselves if they can engage in a action without a medical degree and if the answer is a “no”, the family physician should be careful and proceed with caution. Critical examination should be done in terms of positives and negatives of acting as a physician and family caregiver.
Klugman, Craig M United States (USA) 2008	Qualitative	To present a first-person narrative of a bioethicist and his experiences caregiving for his elderly aunt post stroke.	Bioethicist N = 1 Gender/Sex: Male Age: NR	The narrative notes that Aunt Rose assisted the bioethicist in becoming a better human being; the opportunity assisted in his ability to serve as a patient advocate for a good death. He became more aware of grief work and counselling professions do with their roles to support families and caregiver.
McConnell, E A United States (USA) 1996	Qualitative	To present a first-person narrative written by a nurse about her interaction with the daughter of a client who is also a nurse.	Nurse N = 1 Gender: Female Age: NR	In terms of nurse’s narrative, she mentioned that in caring for her own mother, she was able to see the importance of treating her patients and their family like her own. It is key to provide support to the family caregiver in terms of providing good nursing care.
Mills, Jayne; Aubeeluck, Aimee United Kingdom 2006	Qualitative	To examine the information needs, available support systems, and impact of the experience in caring for a relative with a life-threatening illness has on nurses and their quality of life and to better understand the impact of nurses in their dual role.	Nurses N = 5 Gender/Sex: NR Age: NR	Results from the study concludes that the study will help with developing consistent family-friendly policies. Further research can help with the change to allow more leave to support family caregivers (equity within workplace). Because of the abundance of negative consequences nurses experience within their dual role, conducting future research may support in providing education to other professionals.
Moskop, John C United States (US) 2018	Qualitative	To examine physicians’ experiences as both a practicing physician and family caregiver while caring for a critically ill family member, to uncover experiences and common themes.	Physicians N = 12 Gender/Sex: NR Age: NR	Practicing physician went through strong emotions when making treatment decisions and when deciding how to best employ their professional role while taking care of their loved ones. Drawing the line between refusing and accepting the request for caring for family members pushed a big challenge for the physicians.
Quinney, Loretto; Dwyer, Trudy; Chapman, Ysanne Australia 2018	Qualitative	To describe the lived experience of being a nurse when caring for a family member through acute exacerbations of chronic illness.	Nurses: N = 15 Gender/Sex: NR Age: NR	Nurses mentioned that responsibilities were worse due to personal relationships, raising expectations to provide care. Knowledge and competency related to disease treatment added stress in terms of partaking in family members’ care. Both roles, as a nurse and as a nursed taking care of family member, is stressful and rewarding.
Ross, M; Rideout, E; Carson, M Canada 1994	Mixed methods	The objective of this study was to examine the experience of nurses whose personal and professional careers both center on the provision of care to others.	Participants: Nurses N = 40 Gender/Sex: 100% Female Age: 25–64,.	Participants felt: 1) high level of satisfaction with their work lives and competent and confident in terms of provision of care and derived pleasure from patients, 2) low level of satisfaction at work because of interpersonal conflicts with supervisors and not satisfied with the organizational dimensions of their work lives, 3) high level of satisfaction with home lives and referred to their partners as very supportive and helpful, 4) high degree of stress, situational crises such as illness or death in family, problematic relationships with family members, financial issues, and heavy care needed by young kids or older/disabled parents.
Simone, C; Seidenschmid, M Canada 1997	Qualitative	To present a third person narrative on a physician regarding his experiences with the hospital care of his wife after she suffered a debilitating stroke and if their perspective on whether a physician who is a family member should be excluded from a loved one’s care.	Physician N = 1 Gender/Sex: Male Age: NR	Healthcare workers are discriminated against for being involved in a family caregiving because of their expertise and ability to understand treatment. It should be a choice for practitioners to support an ill family member. Narrative suggests that family members who have the role of a family members should not have to be excluded from taking care of their loved one.
Stocker, S United States (USA) 1996	Qualitative (design not specified) and no methods described. First person narrative.	To provide advice to other nurses who are caregivers to their aging parents. Outlines six ways to reduce caregiver burden and incorporating caregiving responsibilities in one’s schedule.	Nurse N = 1 Gender/Sex: Female Age: NR	It is difficult to play the role of caregiver while caring for your own child. However, by acknowledging changes that are occurring and taking correct steps to deal with changes, individuals can better be prepared as family caregiver and can take on challenges and physical burdens that caregiving can throw.
Wald, Hedy S United States (USA) 2019	Qualitative.	To present a first-person narrative by a clinical professor and her experiences with caregiving for her husband (a neurologist) with glioblastoma.	Nurse N = 1 Gender/Sex: Female Age: NR	It is identified that resilience is a must for success as a caregiver of a family member who has a terminal illness. As a clinical professor, she recognizes herself as a family caregiver and uses her experience in taking care of her dying husband to inform her work.
Ward-Griffin, Catherine Canada 2004	Qualitative.	To discuss the challenges faced by women who provide care in both their work their family lives ("double-duty caregivers")	Nurses N = 15 Gender/Sex: 100% Female Age: 23–64	Nurses who is a family caregiver to an older relative, must negotiate boundaries that come between their professional and personal life as a caregiver. Gender ideologies was seen to have a huge and direct impact on women’s lives. It was concluded that better supportive policies and practices within workplace and society are needed to sustain the health of family caregivers and those receiving care.
Wilson, Kathleen B; Ardoin, Katherine B United States (USA) 2013	Qualitative.	To present first-person narratives describing the personal and professional roles encountered by two nurse-daughters during the end-of-life journey with their respective parent.	Nurses: N = 2 Gender/Sex: Female Age: NR	It was found that when nurses encounter others who are also nurses that are going through similar experience, they utilize their experiences to assist them with coping. Therefore, the importance of family centered care surfaced which can help assist nurses in terms of understanding that nurse-family members are valuable team members within the care process, rather than an opponent.

### Findings

Thematic analysis resulted in four themes– 1) Expectations Related to Performing the Dual Roles of Health Care Professional and Family Caregiver, 2) Understanding the Personal Consequences of Providing Care, 3) Positive Aspects of Dual Roles and 4) Support Needs.

### Expectations related to performing the dual roles of health care professional and family caregiver (HCPC)

Expectations played an important role in shaping the experiences of HCPCs as they were performing the dual roles of health care professional and family caregiver. These expectations were multifaceted and stemmed from various sources, including personal beliefs and professional backgrounds, as well as external pressures from family members and formal care teams. Health care professionals often felt obligated to adopt caregiving roles because of their professional training and experience. Caregiving roles usually included being an advocate for their relative by acting as a liaison and communicating with the health care team, providing hands on medical care, and providing surrogate decision making [32–39]. Most articles contributed to this theme (n = 16, 84%).

The expectations that HCPCs place upon themselves were often driven by healthcare professional training, family values, and a strong sense of responsibility toward aging family members. HCPCs had difficulty separating their medical knowledge and professional expectations from their family caregiving roles [33, 34, 37, 40]. The double-duty caregiving framework includes "caring about" (affection) and "caring for" (meeting needs) [38]. It generates four storylines: family care and nursing care in both dimensions. Healthcare professionals (HCPCs) often struggle to balance emotional involvement with caregiving duties in both family and professional settings [38]. This struggle challenged HCPCs who were expected by other professionals to practice emotional distancing and objectivity in medical decision making [33, 35, 36, 39–41]. For some, assuming caregiving roles was seen as a familial obligation they willingly embraced [32, 34–36, 38]. Some HCPCs internalized reciprocity as a family value, which led them to embrace caregiving roles [32]. In some situations, HCPCs described feeling expected to fill gaps in care not being undertaken by other family [32, 34, 38] and to put their family care before their career [42]. In other situations, HCPCs felt helpless in the caregiving role, struggling with their own expectations that they should be able to help their family members beyond their capacities [33, 38]. This resulted in HCPCs struggling to separate their identity as a healthcare-professional from a family caregiver [36, 43].

HCPCs also faced expectations imposed by healthcare professionals involved in their family member’s care. Expectations included being able to seamlessly navigate a personal caregiving role while upholding the standards and responsibilities associated with their profession. Others’ expectations seem to come from the knowledge about service delivery and medicine that healthcare professionals who were caregivers were expected to have [32–38, 43, 44]. Yet, sometimes HCPCs were perceived by other (non-caregiving) healthcare professionals within the care team as being overly controlling or domineering in their approach to providing care for their family members. At times, the professional care team felt it would be beneficial for the HCPCs to take a less active role in caregiving to create a more balanced and collaborative caregiving environment [41, 45]. Some members of the professional care team also anticipated that HCPCs would be biased in their medical decision making and therefore the professional care team conflicted in how to best treat the patient [34, 40].

Occasionally, ethical boundaries between healthcare professionals and family were noted [35, 36, 38, 40]. For example, upon learning that there was a healthcare professional among the family caregivers, the treating physician would tell the patient to call their family member, rather than refer them to specialist care [40]. This expectation for HCPCS may have arisen to alleviate the burden on the healthcare team who were often understaffed or challenged within the healthcare infrastructure [32]. Sometimes physicians would share private information with the HCPCs that was typically not shared with families [32, 33].

HCPCs often faced family expectations to take on prominent caregiving roles. HCPCs often did not feel that they had a choice not to assume a caregiving role [31, 35, 39]. Family members commonly overlooked the complexities and challenges of balancing dual roles. One study described caregivers as being expected by other members in the family to care for an aging family member to reciprocate the care a parent once provided to them [32]. All family members believed HCPC could use their professional status and access to healthcare resources to better advocate for their aging family member’s care needs [32, 43]. However, some HCPCs did not have as much knowledge as family members expected [32] and experienced challenges in effectively meeting their families’ expectations [33]. Moreover, HCPCs were expected to share their knowledge and skills with family members [32–35] and expected to readily offer their healthcare services to their care recipient while at home [38].

Gender significantly influenced the experience of healthcare professionals (HCPCs) in caregiving roles. Male HCPCs were often assigned a managerial role, delegating hands-on caregiving tasks to female family members, while addressing health-related queries and advocating for healthcare resources [32, 35];. In contrast, female caregivers, particularly in nursing, were expected to balance both emotional and physical caregiving responsibilities alongside their professional duties [33, 46]. Female nurses expressed feelings of caregiving without adequate support from other family members [46]. Male HCPCs, including nurses, faced the challenge of prioritizing their professional role over traditional gendered expectations associated with caregiving [32, 38];. Conversely, female healthcare professionals navigated the dual demands of work and caregiving, especially in female-dominated professions like nursing [33, 38]. These findings underscore the complex interplay of gender dynamics within caregiving roles and healthcare professions, highlighting the need for tailored support and recognition of gender-specific challenges in caregiving contexts.

### Understanding the personal consequences of providing care

This theme was common and described HCPCs perceptions that they often neglected their own health because of their dual roles of healthcare professional and family caregiver (n = 17, 89%). Despite some HCPCs feeling prepared to take on caregiving roles, HCPCs still experienced stress and guilt [32, 33, 40]. Stress sometimes arose because HCPCs hid health information about their relative from other family members [33]. Additionally, they reported physical consequences of caregiving like back pain from lifting both patients and family members [42]. Some HCPCs struggled with healthy eating, finding time to socialize, and getting adequate sleep and exercise [42, 43, 47]. Participants also described balancing work and family caregiving as contributing to burnout [31, 36, 42, 46, 48]. In one study, working while sleep deprived was reported as leading to increased errors at work [47].

Several factors mitigated the negative impact of caregiving on HCPCs. Some participants described professional skills like proactive problem solving as contributing to their preparation for their caregiving role [32, 39]. By problem-solving, HCPCs alleviated some negative personal health consequences associated with juggling their roles [32, 39].

### Dual roles can have a positive impact on providing care

Holding dual roles sometimes enhanced the quality and effectiveness of care provision related to both family and professional caregiving activities (n = 7, 54%). Working and caregiving provided HCPCs with personal satisfaction, despite sometimes modifying work schedules (e.g., working part-time) [42]. HCPCs regularly drew parallels between the care they provided to their family members and the care they offered to their patients throughout the caregiving process [38]. For example, HCPCs reported treating their patients with a level of care and empathy akin to how they would care for their own family members [45]. HCPCs also became more supportive of the fellow family caregivers within their professional circles [35, 49]. One professor began teaching narrative medicine, drawing inspiration from her personal journey of caring for her husband [49]. [38] HCPCs also described their family caregiving as contributing to a sense of reward and resiliency in their personal life [49].

HCPC’s healthcare knowledge and skills often served as a source of reassurance for their family members and played a pivotal role in enhancing the quality of care provided, ultimately contributing to better patient outcomes. Other family members often took comfort in knowing that HCPCs had the skills and insights needed to provide high-quality care and support to their aging family member [43, 49]. This knowledge benefited and improved the care HCPCs provided, and likely positively impacted the patient’s health [32]. Moreover, many HCPCs highlighted how their connections and positions in the healthcare system contributed to their family member receiving better care [32, 33, 49]. For example, some studies found that being a nurse allowed HCPCs to determine and secure the appropriate level of care for their family member from home care services and professionals [32, 33]. Lastly, HCPCs leveraged their medical training to obtain intimate knowledge about their family member’s health status, that in turn, facilitated informed care provision [33].

### HCP caregivers’ needs for support in their dual role

Participants discussed several support needs and sources of support (n = 7, 37%). Healthcare professionals wanted to sustain their paid employment, while also providing care to an aging family member [38, 42]. With appropriate “family-friendly” [43] workplace accommodations and support, healthcare professionals believed they could balance both roles [32, 38, 42, 46, 47]. Workplace accommodations, such as flexibility in adjusting work schedules, and part-time work, made participants feel supported by their organization [38, 42, 47]. When accommodations were not available, some HCPCs had to take leaves of absence [42] or change jobs [47].

HCPCs emphasized the importance of support from other healthcare professionals. This teamwork fostered a strong sense of camaraderie, which was instrumental in effectively managing their dual roles [38, 42]. One study described healthcare professional colleagues providing hands-on care to the HCPC’s parent, when the HCPC was not available [32]. Healthcare professional peers were also seen as understanding the unpredictable commitment of being a family caregiver, which had implications for fostering supportive workplace environments and peer relationships [42, 47].

Beyond personal workplace assistance, HCPCs expressed a strong desire for additional support for their relative from the healthcare system, including their peers [33]. For example, HCPCs described desiring more compassionate physical and mental health care for their family member [33]. Other HCPCs described being concerned by the lack of continuity of care, particularly needing to continually repeat information to healthcare professionals, which could result in tensions and a questioning of the quality of care being provided [33].

## Discussion

This paper adds to our understanding of caregiving by presenting findings from a scoping review of 19 studies on HCPCs’ experiences and support needs. We identified 4 themes related to expectations, the personal consequences of care, positive impact of dual roles, and support needs. These finding underscore the critical need for heightened awareness and tailored support measures for healthcare professionals who serve as family caregivers. Our findings offer insights that are relevant to healthcare workers, organizations, and researchers, by delineating avenues for targeted policies, practices, and future research to enhance the well-being of this unique demographic.

This review highlighted expectations to provide care from the caregiver themselves, other family members, and members of their family members’ care team. HCPCs highlighted the stress they feel from perceived excessive demands. Families largely anticipated HCPCs to significantly contribute to most caregiving duties. Moreover, stress was sometimes heightened by interactions with other healthcare professionals perceived as adversarial or confrontational. The non-health care professional caregiving literature discusses caregiving expectations (e.g., related to caregiver gender) [50]. This review highlights, in addition to gender expectations, the unique expectations that HCPCs encounter due to the overlap between their professional and family caregiver roles. Non-HCP caregivers have noted similar expectations related to their own personal values in taking the caregiving role, managing emotional care and treatment adherence that led to moral distress when unable to fulfill their expectations [51–53]. Similarly, working caregivers often have to take on caregiving roles related to their professional role, such as lawyers providing support with legal issues [54, 55]. Furthermore, examining themes that emerge in populations of health professions beyond nursing could elucidate profession-specific challenges and support needs, contributing to a more comprehensive understanding of caregiving experiences across the healthcare workforce. By examining how physicians, bioethicists, and potentially other professionals navigate their dual roles as caregivers, we can deepen our understanding of the multifaceted nature of caregiving across the healthcare spectrum. This comparative analysis may unveil profession-specific factors, such as training backgrounds, organizational cultures, and role expectations, shaping caregiving experiences, thereby informing tailored interventions and support strategies for diverse healthcare professionals engaged in caregiving responsibilities.

Within the literature review, we found that HCPCs who identify as men and women often assumed roles that are common in the general caregiving literature [56, 57]. That is, women HCPCs not only took on managerial oversight of caregiving, but also hands-on tasks. Male HCPCs often had managerial oversight with less hands-on care.

The personal consequences of caregiving are multifaceted and include challenges to maintaining workflow. Comparatively, these outcomes often align with the broader patterns observed in general caregiving literature [58–62]. Uniquely, HCPCs outline consequences on their healthcare careers. HCPC’s discuss their needs for workplace accommodations including flexible work scheduling, job modifications, part-time options, and adjusted hours [38, 42, 47]. The non-HCP literature suggests caregivers sometimes worried about maintaining their professional status, and the fear of the negative impact on career progression [63]. While workplaces are recognized as crucial for supporting working caregivers [64], HCPCs have often been overlooked in existing studies [65, 66]. Thus, it is important to educate employers, managers, and co-workers within healthcare on strategies to support HCP colleagues with caregiving responsibilities. Additionally, further research is needed to explore not only additional supports that may be beneficial, but also disclosure decisions. Research elsewhere suggests that many workers prefer not to disclose personal information to keep personal information private, which can have implications for support provision [67]. Research is crucial to comprehend disclosure variations among diverse health professional occupations, examining influences like gender, age, race, and culture, linked to concerns about reputational damage and maintaining personal-professional boundaries, notably among female HCPs [68].

Future research in this domain should address several critical aspects. First, it should address methodological quality to ensure robust and reliable findings. Future research should aim to investigate gender differences along the caregiving trajectory, which may further be influenced by their relationship to the care recipient [69]. This could be accomplished through longitudinal qualitative interviews, allowing for a deeper understanding of how gender roles evolve over time within the caregiving context. Additionally, incorporating additional quantitative research methods, such as a survey of HCPC, could provide complementary insights, especially considering the limited number of studies employing these methods as found in this review. Second, there is a pressing need to explore the themes identified in this review in more depth. For example, qualitative interviews with diverse HCPC samples and semi-structured interviews may capture rich data to further refine our understanding of support needs and the impact on one’s health. In this review, most research explored the caregiving situation at a single point in time, and thus, research exploring the lived experiences of HCPCs should consider changes across the caregiving trajectory by gender and relationship [70]. Researchers should delve into issues related to sampling and rigorously report sample characteristics, with a particular emphasis on the role of gender in caregiving experiences [69]. Mixed methods approaches using standardized measurement approaches, such as the Caregiver Assistant Scale [71], in conjunction with qualitative data collection can help provide a more multi-facetted understanding of HCPCs’ experiences. Likewise, the Double Duty Caregiver Scale [72] offers a valuable tool for quantitatively assessing the experiences of healthcare professionals who are also family caregivers. This scale allows for the measurement of various dimensions of double-duty caregiving, providing insights into the prevalence, frequency, and perceived burden of caregiving responsibilities among this population [72].

### Limitations

The current scoping review has several limitations. We excluded grey literature to focus on academic research for informing future empirical studies. Our search was also limited to English-language studies, potentially excluding valuable insights from other cultures and jurisdictions and limiting the generalizability of our findings to non-English speaking HCPCs. In this study, we focused on regulated health providers and caregivers of older adults to maintain a specific scope and depth of analysis. We acknowledge that this focus excludes non-regulated health providers and caregivers of other age groups, potentially overlooking a significant segment of double-duty caregivers. Future research should aim to include a broader range of caregivers to provide more comprehensive insights into the experiences of double-duty caregivers.

## Conclusion

This review serves as a foundational exploration of HCPCs’ experiences, shedding light on the imperative to enhance their well-being amid multifaceted caregiving roles, and their need for additional healthcare system support. With a focus on expectations shaping dual roles, our findings reveal the intricate interplay of professional and familial obligations. Healthcare professionals, often driven by internal and external expectations, navigate complex caregiving dynamics, impacting their own well-being. The review sheds light on the nuanced personal consequences, positive impacts, and identified support needs, emphasizing the necessity for tailored workplace accommodations and collaborative healthcare system support to sustain the critical dual roles of healthcare professionals as family caregivers. Encouraging further intersectional research is vital for understanding and sustaining the dual caregiving roles of healthcare professionals.

## Supporting information

S1 Checklist Prisma-SCr. (PDF)

S1 File Search strategy. (DOCX)

S2 File Codebook. (DOCX)

S3 File (DOCX)
