# Correction: A scoping review to examine health care professionals’ experiences as family caregivers

**DOI:** 10.1371/journal.pone.0320458

**Published:** 2025-03-18

**Authors:** Kristina M. Kokorelias, Nira Rittenberg, Orianna Scali, Suzanne Smith-Bayley, Monique A. M. Gignac, Gary Naglie, Jessica E. Shiers, Jill I. Cameron

The seventh author’s name is spelled incorrectly. The correct name is: Jessica E. Shiers
