# Supplementary material for: A scoping review to examine health care professionals’ experiences as family caregivers
Source: PLoS One. 2025 Jan 22;20(1):e0308657. doi: 10.1371/journal.pone.0308657 (PMC11753689; doi:10.1371/journal.pone.0308657)
Supplement: S1 File — (DOCX) [file pone.0308657.s002.docx]

**Search Strategy and Supporting Documentation**

**SEARCH DETAILS**

Searches were conducted in MEDLINE(R) (in Ovid, including Epub Ahead of Print, In-Process & Other Non-Indexed Citations, Ovid MEDLINE(R) Daily), Embase (Ovid), APA PsycInfo (Ovid), AMED (Ovid), and CINAHL (EBSCO). Searches were conducted in January 2021.

Search strategies included the use of keywords and subject headings (e.g. MeSH, Emtree) related to the ethics of physicians and health care workers treating immediate family. Searches were limited to human populations, and English language records. Searches were developed and conducted by an Information Specialist (JH) with feedback from the research team.

**PRE-DUPLICATE REMOVAL RESULTS (for PRISMA Reporting)**

*TOTAL Results: 2253 citations before de-duplication*

- Medline: 1477 citations
- Embase: 354 citations
- APA PsycInfo: 323 citations
- AMED: 89 citations
- CINAHL: 10 citations

**SEARCH STRATEGIES**

- - ** subject heading allied health personnel accounts for the following:
  - allied health personnel
  - allied health professional
  - allied health professionals
  - assistant, healthcare
  - assistants, healthcare
  - health personnel, allied
  - health professional, allied
  - health professionals, allied
  - healthcare assistant
  - healthcare assistants
  - healthcare support worker
  - healthcare support workers
  - paramedic
  - paramedical personnel
  - paramedics
  - personnel, allied health
  - personnel, paramedical
  - population program specialist
  - population program specialists
  - professional, allied health
  - professionals, allied health
  - program specialist, population
  - program specialists, population
  - specialist, population program
  - specialists, population program
  - support worker, healthcare
  - support workers, healthcare
  - worker, healthcare support
  - workers, healthcare support

**Database: Ovid MEDLINE(R) ALL <1946 to January 08, 2021>**

Search Strategy:

--------------------------------------------------------------------------------

| 1 | \| allied health personnel.mp. or exp Allied Health Personnel/ \| \| --- \| | 50484 |
| --- | --- | --- | --- |
| 2 | \| exp Caregivers/ or caregiving.mp. \| \| --- \| \| | 44028 |
| 3 | \| 1 and 2 \| \| --- \| | 384 |
| 4 | \| exp Physicians, Primary Care/ or exp Osteopathic Physicians/ or exp Physicians, Family/ or exp Occupational Health Physicians/ or exp Physicians/ or exp Physicians, Women/ \| \| --- \| \| | 146307 |
| 5 | \| 2 and 4 \| \| --- \| \| | 530 |
| 6 | \| exp Physician Assistants/ \| \| --- \| \| | 5838 |
| 7 | \| 2 and 6 \| \| --- \| \| | 5 |
| 8 | \| exp Occupational Therapists/ \| \| --- \| \| | 355 |
| 9 | \| exp Physical Therapists/ \| \| --- \| \| | 2040 |
| 10 | \| exp Nurses/ \| \| --- \| \| | 89407 |
| 11 | \| exp Midwifery/ \| \| --- \| \| | 19366 |
| 12 | \| exp Dentists/ \| \| --- \| \| | 19359 |
| 13 | \| exp Social Workers/ \| \| --- \| \| | 694 |
| 14 | \| exp Medical Laboratory Personnel/ \| \| --- \| \| | 2698 |
| 15 | \| exp Optometrists/ \| \| --- \| \| | 95 |
| 16 | \| exp Nutritionists/ \| \| --- \| \| | 1279 |
| 17 | \| exp Physiatrists/ \| \| --- \| \| | 60 |
| 18 | \| 2 and 8 \| \| --- \| \| | 8 |
| 19 | \| 2 and 9 \| \| --- \| \| | 20 |
| 20 | 2 and 10 | 541 |
| 21 | 2 and 11 | 33 |
| 22 | 2 and 12 | 23 |
| 23 | 2 and 13 | 36 |
| 24 | 2 and 14 | 2 |
| 25 | 2 and 15 | 0 |
| 26 | 2 and 16 | 3 |
| 27 | 2 and 17 | 0 |
| 28 | 3 or 5 or 7 or 18 or 19 or 20 or 21 or 22 or 23 or 24 or 25 or 26 or 27 | 1477 |

Embase

| 1 | \| allied health personnel.mp. or exp Allied Health Personnel/ \| \| --- \| | 530568 |
| --- | --- | --- | --- |
| 2 | \| exp Caregivers/ or caregiving.mp. \| \| --- \| \| | 92348 |
| 3 | \| 1 and 2 \| \| --- \| | 9132 |
| 4 | \| exp Physicians, Primary Care/ or exp Osteopathic Physicians/ or exp Physicians, Family/ or exp Occupational Health Physicians/ or exp Physicians/ or exp Physicians, Women/ \| \| --- \| \| | 831810 |
| 5 | \| 2 and 4 \| \| --- \| \| | 8446 |
| 6 | \| exp Physician Assistants/ \| \| --- \| \| | 8477 |
| 7 | \| 2 and 6 \| \| --- \| \| | 60 |
| 8 | \| exp Occupational Therapists/ \| \| --- \| \| | 7500 |
| 9 | \| exp Physical Therapists/ \| \| --- \| \| | 22934 |
| 10 | \| exp Nurses/ \| \| --- \| \| | 193416 |
| 11 | \| exp Midwifery/ \| \| --- \| \| | 33862 |
| 12 | \| exp Dentists/ \| \| --- \| \| | 28776 |
| 13 | \| exp Social Workers/ \| \| --- \| \| | 13639 |
| 14 | \| exp Medical Laboratory Personnel/ \| \| --- \| \| | 343 |
| 15 | \| exp Optometrists/ \| \| --- \| \| | 816 |
| 16 | \| exp Nutritionists/ \| \| --- \| \| | 13077 |
| 17 | \| exp Physiatrists/ \| \| --- \| \| | 598 |
| 18 | \| 2 and 8 \| \| --- \| \| | 411 |
| 19 | \| 2 and 9 \| \| --- \| \| | 593 |
| 20 | 2 and 10 | 5096 |
| 21 | 2 and 11 | 209 |
| 22 | 2 and 12 | 130 |
| 23 | 2 and 13 | 1098 |
| 24 | 2 and 14 | 2 |
| 25 | 2 and 15 | 8 |
| 26 | 2 and 16 | 3 |
| 27 | 2 and 17 | 18 |
| 28 | 3 or 5 or 7 or 18 or 19 or 20 or 21 or 22 or 23 or 24 or 25 or 26 or 27 | 15503 |
| 29 | immediate family.mp. | 1417 |
| 30 | 28 and 29 | 13 |
| 31 | exp extended family/ or exp nuclear family/ or exp family relation/ or exp family decision making/ or exp family assessment/ or exp family/ or exp family interaction/ or exp family conflict/ | 577948 |
| 32 | 29 or 31 | 578881 |
| 33 | 28 and 32 | 2605 |
| 34 | limit 33 to aged <65+ years> | 354 |

APA PsycInfo

| 1 | \| allied health personnel.mp. or exp Allied Health Personnel/ \| \| --- \| | 6410 |
| --- | --- | --- | --- |
| 2 | \| exp Caregivers/ or caregiving.mp. \| \| --- \| \| | 36357 |
| 3 | \| 1 and 2 \| \| --- \| | 172 |
| 4 | \| exp Physicians, Primary Care/ or exp Osteopathic Physicians/ or exp Physicians, Family/ or exp Occupational Health Physicians/ or exp Physicians/ or exp Physicians, Women/ \| \| --- \| \| | 44241 |
| 5 | \| 2 and 4 \| \| --- \| \| | 375 |
| 6 | \| exp Physician Assistants/ \| \| --- \| \| | 0 |
| 7 | \| 2 and 6 \| \| --- \| \| | 0 |
| 8 | \| exp Occupational Therapists/ \| \| --- \| \| | 2310 |
| 9 | \| exp Physical Therapists/ \| \| --- \| \| | 530 |
| 10 | \| exp Nurses/ \| \| --- \| \| | 32501 |
| 11 | \| exp Midwifery/ \| \| --- \| \| | 1430 |
| 12 | \| exp Dentists/ \| \| --- \| \| | 469 |
| 13 | \| exp Social Workers/ \| \| --- \| \| | 12672 |
| 14 | \| exp Medical Laboratory Personnel/ \| \| --- \| \| | 0 |
| 15 | \| exp Optometrists/ \| \| --- \| \| | 121 |
| 16 | \| exp Nutritionists/ \| \| --- \| \| | 0 |
| 17 | \| exp Physiatrists/ \| \| --- \| \| | 0 |
| 18 | \| 2 and 8 \| \| --- \| \| | 42 |
| 19 | \| 2 and 9 \| \| --- \| \| | 8 |
| 20 | 2 and 10 | 689 |
| 21 | 2 and 11 | 19 |
| 22 | 2 and 12 | 2 |
| 23 | 2 and 13 | 227 |
| 24 | 2 and 14 | 0 |
| 25 | 2 and 15 | 0 |
| 26 | 2 and 16 | 0 |
| 27 | 2 and 17 | 0 |
| 28 | 3 or 5 or 7 or 18 or 19 or 20 or 21 or 22 or 23 or 24 or 25 or 26 or 27 | 1404 |
| 29 | immediate family.mp. | 751 |
| 30 | 28 and 29 | 3 |
| 31 | exp extended family/ or exp nuclear family/ or exp family relation/ or exp family decision making/ or exp family assessment/ or exp family/ or exp family interaction/ or exp family conflict/ | 306732 |
| 32 | 29 or 31 | 307138 |
| 33 | 28 and 32 | 323 |

AMED

| 1 | \| allied health personnel.mp. or exp Allied Health Personnel/ \| \| --- \| | 662 |
| --- | --- | --- | --- |
| 2 | \| exp Caregivers/ or caregiving.mp. \| \| --- \| \| | 3425 |
| 3 | \| 1 and 2 \| \| --- \| | 14 |
| 4 | \| exp Physicians, Primary Care/ or exp Osteopathic Physicians/ or exp Physicians, Family/ or exp Occupational Health Physicians/ or exp Physicians/ or exp Physicians, Women/ \| \| --- \| \| | 44241 |
| 5 | \| 2 and 4 \| \| --- \| \| | 375 |
| 6 | \| exp Physician Assistants/ \| \| --- \| \| | 0 |
| 7 | \| 2 and 6 \| \| --- \| \| | 0 |
| 8 | \| exp Occupational Therapists/ \| \| --- \| \| | 2310 |
| 9 | \| exp Physical Therapists/ \| \| --- \| \| | 530 |
| 10 | \| exp Nurses/ \| \| --- \| \| | 32501 |
| 11 | \| exp Midwifery/ \| \| --- \| \| | 1430 |
| 12 | \| exp Dentists/ \| \| --- \| \| | 469 |
| 13 | \| exp Social Workers/ \| \| --- \| \| | 12672 |
| 14 | \| exp Medical Laboratory Personnel/ \| \| --- \| \| | 0 |
| 15 | \| exp Optometrists/ \| \| --- \| \| | 121 |
| 16 | \| exp Nutritionists/ \| \| --- \| \| | 0 |
| 17 | \| exp Physiatrists/ \| \| --- \| \| | 0 |
| 18 | \| 2 and 8 \| \| --- \| \| | 42 |
| 19 | \| 2 and 9 \| \| --- \| \| | 8 |
| 20 | 2 and 10 | 689 |
| 21 | 2 and 11 | 19 |
| 22 | 2 and 12 | 2 |
| 23 | 2 and 13 | 227 |
| 24 | 2 and 14 | 0 |
| 25 | 2 and 15 | 0 |
| 26 | 2 and 16 | 0 |
| 27 | 2 and 17 | 0 |
| 28 | 3 or 5 or 7 or 18 or 19 or 20 or 21 or 22 or 23 or 24 or 25 or 26 or 27 | 1404 |
| 29 | immediate family.mp. | 751 |
| 30 | 28 and 29 | 3 |
| 31 | exp extended family/ or exp nuclear family/ or exp family relation/ or exp family decision making/ or exp family assessment/ or exp family/ or exp family interaction/ or exp family conflict/ | 306732 |
| 32 | 29 or 31 | 307138 |
| 33 | 28 and 32 | 323 |

***************************

**Database: CINAHL Complete (EBSCO) –**

| S12 | S6 AND S10 | Expanders - Apply equivalent subjects Search modes - Boolean/Phrase | 3 |
| --- | --- | --- | --- |
| S11 | S9 AND S10 | Expanders - Apply equivalent subjects Search modes - Boolean/Phrase | 7 |
| S10 | ""treat* family"" OR (MH "Professional-Family Relations/EI") | Expanders - Apply equivalent subjects Search modes - Boolean/Phrase | 421 |
| S9 | S5 AND S7 | Narrow by SubjectMajor: - conflict of interest Narrow by SubjectMajor: - physician's role Narrow by SubjectMajor: - medical practice Narrow by SubjectMajor: - professional practice Narrow by SubjectMajor: - physician-patient relations Narrow by SubjectMajor: - ethics, medical Search modes - Boolean/Phrase | 1287 |
| S8 | S5 AND S7 | Expanders - Apply equivalent subjects Search modes - Boolean/Phrase | 7551 |
| S7 | S1 OR S4 OR S6 | Expanders - Apply equivalent subjects Search modes - Boolean/Phrase | 76920 |
| S6 | (MH "Family Relations+/EI") | Expanders - Apply equivalent subjects Search modes - Boolean/Phrase | 50 |
| S5 | S2 OR S3 | Expanders - Apply equivalent subjects Search modes - Boolean/Phrase | 76431 |
| S4 | (MH "Codes of Ethics") OR (MH "Ethics, Medical") OR (MH "Ethics, Professional") | Expanders - Apply equivalent subjects Search modes - Boolean/Phrase | 16258 |
| S3 | (MH "Allied Health Personnel") | Expanders - Apply equivalent subjects Search modes - Boolean/Phrase | 4344 |
| S2 | (MH "Physicians") OR (MH "Physician Assistants") OR (MH "Medical Staff, Hospital") | Expanders - Apply equivalent subjects Search modes - Boolean/Phrase | 72453 |
| S1 | (MH "Physician Attitudes") OR (MH "Physician Assistant Attitudes") OR (MH "Attitude of Health Personnel") | Expanders - Apply equivalent subjects Search modes - Boolean/Phrase | 61489 |
