# Supplementary material for: A scoping review to examine health care professionals’ experiences as family caregivers
Source: PLoS One. 2025 Jan 22;20(1):e0308657. doi: 10.1371/journal.pone.0308657 (PMC11753689; doi:10.1371/journal.pone.0308657)
Supplement: S2 File — (DOCX) [file pone.0308657.s003.docx]

**Work environment/context** – situations where participants describe how aspects of their work is related to their caregiving experience.

**Timing**– when participants discuss aspects of the timing of their experiences in the context of the illness trajectory

**Family centered care** – any reference to family involvement in care in relation to the care delivery system

**Experience-** any reference to the participants experience in general that does not fit with other codes

**Support Needs**- any reference to the participants’ needs or unmet needs of support including emotional, appraisal, tangible or informational

**Impact of Caregiving on Work**- any reference to the impact caregiving has on a participants work, including education, professional development, education etc.

**Impact of Work on Caregiving**- any reference to the impact a participants healthcare profession, including education and work situation, has on caregiving.

**Benefits of caregiving**- any reference to positive aspects of caregiving (benefits in relation to physical, emotional, social, or any other perceived benefit)

**Communication**- any reference to communicating with healthcare teams, colleagues or other caregivers

**Consequences of caregiving** – any reference to the impact on physical, emotional, social health and well being, or any other perceived consequence (not work as it is included elsewhere)

**MISC**- anything that is not covered by any of the other codes

**Context**- any reference to contextual details about participants (e.g., demographics)

**Clinical implications** – as presented by author in the context of the discussion section

**Future research areas** – as presented by author in the context of the discussion section

**Health system implications** – as presented by author in the context of the discussion section
