# Supplementary material for: A scoping review to examine health care professionals’ experiences as family caregivers
Source: PLoS One. 2025 Jan 22;20(1):e0308657. doi: 10.1371/journal.pone.0308657 (PMC11753689; doi:10.1371/journal.pone.0308657)
Supplement: S3 File — (DOCX) [file pone.0308657.s004.docx]

| **Author/Title of article** | **Is there congruity between the stated philosophical perspective and the research methodology?** | **Is there congruity between the research methodology and the research question or objectives?** | **Is there congruity between the research methodology and the methods used to collect the data?** | **Is there congruity between the research methodology and the representation and analysis of the data?** | **Is there congruity between the research methodology and the interpretation of results?** | **Is there a statement locating the researcher culturally or theoretically?** | **Is the influence of the researcher on the research, and vice-versa, addressed?** | **Are participants, and their voices, adequately represented?** | **Is the research ethical according to current criteria or, for recent studies, and is there evidence of ethical approval by an appropriate body?** | **Do the conclusions drawn in the research report flow from the analysis, or interpretation, of the data?** |
| --- | --- | --- | --- | --- | --- | --- | --- | --- | --- | --- |
| Ross, M M; Rideout, E; Carson, M/Nurses' work: balancing personal and professional caregiving careers. | Yes | Yes | Yes | Yes | Yes | No | N/A | Yes | N/A | Yes |
| Klugman, Craig M/Dual roles of the care provider at the end of life: An autoethnography. | N/A | N/A | N/A | N/A | N/A | Yes | N/A | N/A | N/A | Yes |
| DePasquale, Nicole; Davis, Kelly D; Zarit, Steven H; Moen, Phyllis; Hammer, Leslie B; Almeida, David M/Combining formal and informal caregiving roles: The psychosocial implications of double- and triple-duty care. | Yes | Yes | Yes | Yes | Yes | No | N/A | Yes | N/A | Yes |
| Wald, Hedy S/Helping My Husband Live and Die. | N/A | N/A | N/A | N/A | N/A | Yes | N/A | N/A | N/A | N/A |
| Stocker, S/Six tips for caring for aging parents. | Yes | Yes | N/A | N/A | N/A | No | N/A | N/A | N/A | N/A |
| Simone, C; Seidenschmid, M/Physicians' role in care of loved ones. | Yes | Yes | N/A | N/A | N/A | N/A | N/A | N/A | N/A | N/A |
| Fromme, Erik K; Farber, Neil J; Babbott, Stewart F; Pickett, Mary E; Beasley, Brent W/What do you do when your loved one is ill? The line between physician and family member. | Yes | Yes | N/A | N/A | N/A | N/A | N/A | N/A | N/A | N/A |
| Ward-Griffin, Catherine/Nurses as Caregivers of Elderly Relatives: Negotiating Personal and Professional Boundaries. | Yes | Yes | Yes | Yes | Yes | No | N/A | Yes | N/A | Yes |
| McConnell, E A/'The daughter is a nurse'. | Yes | Yes | N/A | N/A | N/A | Yes | N/A | N/A | N/A | N/A |
| Wilson, Kathleen B; Ardoin, Katherine B/When professional and personal worlds meet: nurse as daughter. | Yes | Yes | Yes | Yes | Yes | No | N/A | Yes | N/A | N/A |
| Quinney, Loretto; Dwyer, Trudy; Chapman, Ysanne/Tensions in the personal world of the nurse family carer: A phenomenological approach. | Yes | Yes | Yes | Yes | Yes | N/A | N/A | No | Yes | Yes |
| DePasquale, Nicole; Bangerter, Lauren R; Williams, Jessica; Almeida, David M/Certified nursing assistants balancing family caregiving roles: Health care utilization among double- and triple-duty caregivers. | Yes | Yes | Yes | Yes | Yes | N/A | N/A | Yes | N/A | Yes |
| Anjos, Ana Paula; Ward-Griffin, Catherine; Leipert, Beverly/Understanding gendered expectations and exemptions experienced by male double-duty caregivers: a qualitative secondary analysis. | Yes | Yes | Yes | Yes | Yes | No | N/A | Yes | N/A | Yes |
| Mills, Jayne; Aubeeluck, Aimee/Nurses' experiences of caring for their own family members | Yes | Yes | Yes | N/A | N/A | No | N/A | Yes | N/A | Yes |
| Clendoni, Jill; Walker, Leonie/Juggling nursing and family care. | Yes | Yes | Yes | Yes | Yes | No | N/A | Yes | N/A | Yes |
| Moskop, John C/Doctor in, and for, the Family?: Physicians Reflect on Care for Loved Ones. | Yes | Yes | Yes | Yes | Yes | N/A | N/A | Yes | N/A | Yes |
| Cicchelli, Lisa; McLeod, Deborah/Lived experiences of nurses as family caregivers in advanced cancer. | Yes | Yes | Yes | Yes | Yes | N/A | N/A | Yes | N/A | Yes |
| Clendon J.; Walker L./Nurses as family caregivers - barriers and enablers facing nurses caring for children, parents or both | Yes | Yes | Yes | Yes | Yes | No | N/A | Yes | Yes | Yes |
| Chen F.M.; Feudtner C.; Rhodes L.A.; Green L.A./Role conflicts of physicians and their family members: Rules but no rulebook | Yes | Yes | Yes | Yes | Yes | N/A | N/A | Yes | N/A | Yes |
